# Model-Based Design to Enhance Neotissue Formation in Additively Manufactured Calcium-Phosphate-Based Scaffolds

**DOI:** 10.3390/jfb14120563

**Published:** 2023-12-03

**Authors:** Bingbing Liang, Ehsan Sadeghian Dehkord, Dorien Van Hede, Mojtaba Barzegari, Bruno Verlée, Justine Pirson, Grégory Nolens, France Lambert, Liesbet Geris

**Affiliations:** 1 Biomechanics Research Unit, GIGA In Silico Medicine, University of Liège, 4000 Liège, Belgium; bingbing.liang@uliege.be (B.L.); ehsan.sadeghian@kuleuven.be (E.S.D.); 2 Prometheus, The R&D Division for Skeletal Tissue Engineering, KU Leuven, 3000 Leuven, Belgium; 3 Department of Periodontology Oral Surgery and Implant Surgery, Faculty of Medicine, University Hospital of Liège, 4000 Liège, Belgium; dorien.vanhede@uliege.be (D.V.H.); france.lambert@uliege.be (F.L.); 4 Dental Biomaterials Research Unit, Faculty of Medicine, University of Liège, 4000 Liège, Belgium; 5 Biomechanics Section, Department of Mechanical Engineering, KU Leuven, 3000 Leuven, Belgium; m.barzegari.shankil@tue.nl; 6 Department of Additive Manufacturing, Sirris Liège, 4100 Seraing, Belgium; bruno.verlee@gmail.com; 7 Wishbone SA, 4400 Flemalle, Belgium; justine.pirson@wishbone-biotech.com; 8 Faculty of Medicine, University of Namur, 5000 Namur, Belgium; gregory.nolens@unamur.be

**Keywords:** bone tissue engineering, dental bone regeneration, porous scaffold, biomaterials, 3D printing, computer modeling and simulation, in silico medicine, optimal design, porosity

## Abstract

In biomaterial-based bone tissue engineering, optimizing scaffold structure and composition remains an active field of research. Additive manufacturing has enabled the production of custom designs in a variety of materials. This study aims to improve the design of calcium-phosphate-based additively manufactured scaffolds, the material of choice in oral bone regeneration, by using a combination of in silico and in vitro tools. Computer models are increasingly used to assist in design optimization by providing a rational way of merging different requirements into a single design. The starting point for this study was an in-house developed in silico model describing the in vitro formation of neotissue, i.e., cells and the extracellular matrix they produced. The level set method was applied to simulate the interface between the neotissue and the void space inside the scaffold pores. In order to calibrate the model, a custom disk-shaped scaffold was produced with prismatic canals of different geometries (circle, hexagon, square, triangle) and inner diameters (0.5 mm, 0.7 mm, 1 mm, 2 mm). The disks were produced with three biomaterials (hydroxyapatite, tricalcium phosphate, and a blend of both). After seeding with skeletal progenitor cells and a cell culture for up to 21 days, the extent of neotissue growth in the disks’ canals was analyzed using fluorescence microscopy. The results clearly demonstrated that in the presence of calcium-phosphate-based materials, the curvature-based growth principle was maintained. Bayesian optimization was used to determine the model parameters for the different biomaterials used. Subsequently, the calibrated model was used to predict neotissue growth in a 3D gyroid structure. The predicted results were in line with the experimentally obtained ones, demonstrating the potential of the calibrated model to be used as a tool in the design and optimization of 3D-printed calcium-phosphate-based biomaterials for bone regeneration.

## 1. Introduction

In recent years, bone regeneration strategies have advanced significantly in clinical practice. While autologous bone grafting remains a gold standard due to its minimal risk of immune rejection and disease transmission, it presents drawbacks, including donor site morbidity, a limited donor volume, and shaping difficulties [1,2]. Ideal bone regeneration materials necessitate osteogenesis, osteoconduction, and osteoinduction. Synthetic biomaterials are gaining attraction as bone scaffolds due to the absence of donor site morbidity and due to their favorable biocompatibility, biodegradability, and foreseeable immunological response [3,4,5,6]. Notably, porous scaffolds, particularly those composed of calcium phosphates (CaPs), play a pivotal role in bone tissue regeneration. CaPs, like hydroxyapatite (HAp) and tricalcium phosphate (TCP), exhibit similarities to bone’s inorganic composition (see review by Hou et al. [7]). With the use of additive manufacturing technologies (AMTs), patient-specific implants have become a (clinical) reality [8,9]. Their design is based on a range of considerations, including the printing technology, the material, and the macroscopic mechanical requirements [7]. The design of microscopic properties has typically been dominated by considerations of interconnectivity, porosity, and pore size. However, in recent years, local curvature has been shown to be an important factor in driving bone regeneration [10,11,12]. 

When optimizing scaffold designs, in silico modelling (i.e., the use of computer modelling and simulation) is a key approach to limiting the amount of in vivo testing required, in line with the 3Rs principle (reduce, refine, and replace animal tests), by selecting the most promising designs based on the predictions made by the model. A variety of models of bone regeneration in silico have been proposed in the literature, with most of them corroborated on the basis of historical or animal experiments. In addition, most of these models focus on regeneration without a support structure [13] or on a predefined shape [14,15,16] rather than using the model to select the optimal internal architecture of the structure. On the other hand, earlier approaches aiming for a more objective optimization often focused on optimizing the mechanical properties of the structure without taking into account internal form or biological requirements [17,18]. Due to the recent increase in attention for curvature-based biology in general [19], scaffold research has also turned to local curvature to optimize the internal design of bone substitutes to maximize neotissue formation [20,21,22,23,24,25,26]. In several cases, dedicated validation experiments have been performed, consisting mostly of in vitro cell culture experiments on titanium, hydroxyapatite, or polycaprolactone 2D and 2D+ substrates. 

In this study, we aim to improve the design of calcium-phosphate-based additively manufactured scaffolds by using a combination of in silico and in vitro tools. We build on our prior work related to the curvature-based modeling of neotissue growth in additively manufactured titanium implants [22] in order to account for the effect of the use of active CaP-based biomaterials. To recalibrate the model, we have designed a dedicated in vitro experiment allowing us to evaluate the effect of pore shape and pore size on neotissue growth in scaffolds produced with HAp, TCP, and an HAp–TCP blend (biphasic calcium phosphate, BCP). After running a Bayesian optimization for the model recalibration, we test the predictive capacity of the model by performing a new neotissue formation experiment, both in silico and in vitro, in a 3D gyroid structure. The observed correspondence between the in vitro and in silico results is an indicator of the potential of the model to be used in the design and optimization of more complex 3D bone tissue engineering scaffolds.

## 2. Materials and Methods

### 2.1. In Silico Model

This section describes the setup, implementation, and optimization of the in silico model for curvature-based neo-tissue growth applied to CaP-based biomaterials. The effect of the released ions is not considered explicitly in the developed model but instead is captured by the changes in the overall neotissue growth rate during the model calibration phase. 

#### 2.1.1. Level Set Method

The level set method (LSM) is a mathematical approach for tracking moving interfaces, in which the parameterization of curves and surfaces can be conveniently performed to study the change in the morphology and topology of objects [27]. We have previously used the LSM to implement curvature-based neotissue growth in titanium scaffolds [22], as, amongst other advantages, it can effectively be used to calculate the average curvature as a guiding factor for tissue growth simulations. 

A signed distance function (φ) describes the distance of each node of the desired domain to the interface. The zero iso-surface determines the moving interface. In the current study, the interface divides the computational domain into two subdomains, neotissue, and void space, according to the following definition:

(1) $$\left\{\begin{array}{c} \;\varphi >0\;in\;\Omega_{nt}\\ \varphi <0\;in\;\Omega_{v}\\ \varphi =0\;in\;\Gamma \end{array}\right.$$

with Ω denoting the domain of interest and Ω_nt_ and Ω_v_ denoting the neotissue and void space subdomains, respectively. The interface between Ω_nt_ and Ω_v_ is denoted by Γ. The LSM formalism for tracking the interface moving with the growth velocity, **v_G_**, can be expressed by the convection equation, describing how the level set function, φ, evolves in the entire domain, Ω, over time:

(2) $$\frac{\partial \varphi }{\partial t}+v_{G}\cdot \nabla \varphi =0\;in\;\Omega$$

**Figure 1:**
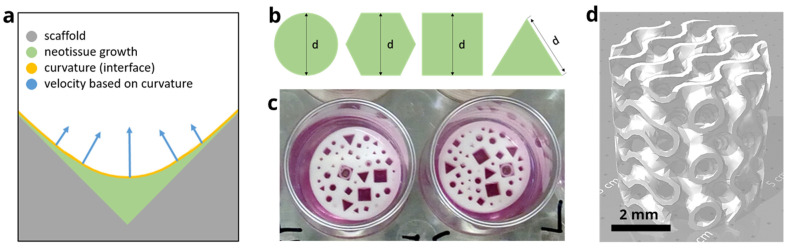
In silico–in vitro experimental design element. (**a**) Schematic representation of the different domains of the level set method showing the curvature-based growth velocity (in blue) as well as the interface (in yellow) between the neotissue (in green, φ > 0) and void space (in white, φ < 0). (**b**) Individual channel geometries and sizes (indicated by d). (**c**) Additively manufactured disks shown in the wells (diameter 14 mm) of a 24-well plate submerged in culture medium. (**d**) 3D scaffold with gyroid design.

This equation is solved with a homogenous Neumann boundary condition (∂φ/∂n=0), with **n** being the normal to the computational domain, Ω. The calculation of the interface advection velocity, ${v_{G}=V_{G}*n}_{\Gamma }$ (with $n_{\Gamma }=\frac{\nabla \varphi }{\left|\nabla \varphi \right|}$), is related to the local mean curvature, κ ($\kappa =\nabla \cdot n_{\Gamma }$), of the neotissue interface (shown in Figure 1a).

$${V_{G}=A\;\cdot g\left(\kappa \right)\cdot n}_{\Gamma }\;g\left(\kappa \right)=\left\{\begin{array}{c} -\kappa ,\;\;if\;\kappa >0\\ 0,\;\;if\;\kappa \leq 0 \end{array}\right.$$

A is a parameter to control the curvature effect, determined from experimental data in a fitting procedure. The negative sign in the definition of g(κ) comes from the fact that, according to our definition of φ, the normal, $n_{\Gamma },$ points towards the neotissue, so growth has to be opposite ∇φ.

#### 2.1.2. Implementation of the Model

The curvature-based model was solved numerically using the finite element method, implemented in the open-source partial differential equation (PDE) solver FreeFEM (v4.6, Laboratoire Jacques-Louis Lions, Université Pierre et Marie Curie, Paris, France) [28]. The computational domain consisted of individual beams of 2 mm height and triangular, squared, hexagonal, or circular cross-sections of diameters 0.5 mm, 0.7 mm, 1 mm, and 2 mm (Figure 1). The geometries and their corresponding computational mesh were created using the SALOME platform (v9.8.0, Salome-platform.org, France) [29], and all the other pre-processing steps were performed in FreeFEM. The computational mesh was generated using a set of first-order tetrahedral elements, and the convergence was checked. 

To initialize the LSM, an initial distance function, φ_0_, was defined in the domain Ω at the boundary of the scaffold. However, the level set function, φ, is not differentiable where the gradient is discontinuous, meaning that the normal, $n_{\Gamma },$ and the curvature, κ, cannot be properly defined everywhere in the domain. A solution is to add a small numerical diffusion term to the expected direction, $n_{\Gamma },$ and curvature, κ. The specific mathematical expression is as follows:

$$n_{\Gamma }=\frac{\nabla \varphi }{\left|\nabla \varphi \right|}+\varepsilon \Delta n_{\Gamma }\;\kappa =\nabla \cdot n_{\Gamma }+\varepsilon \Delta \kappa$$

During the verification process, a comparison of images generated by different diffusion values showed that the smaller the value of ε, the greater the oscillation of the curvature calculation and the worse the smoothness of the boundary. Conversely, the larger the value of ε, the larger the influence of the numerical diffusion, generating erroneous results. In [22], the parameter ε was fixed at 1 × 10^−4^ based on a comparison between the numerical and analytical solutions. In the process of initializing the level set function φ, the open-source software mshdist (v1.0, by Charles Dapogny (Université Joseph Fourier) and Pascal Frey (Université Pierre et Marie Curie), France) [30] was used to avoid the level set distortion that the distance function in the model application may cause.

To reduce the computational cost, the method of characteristics can be used in FreeFEM [31]. This method reduces a partial differential equation (PDE) to a system of ordinary differential equations (ODE) along curves called characteristics. The resolution of these ODEs along those curves leads to the solution of the original PDE. To further improve the performance of the model and decrease the execution time of simulations, model parallelization was taken into account. Parallelization was considered for two main stages of the computation pipeline: assembling the matrices and solving the resulting linear system of equations. As part of a standard finite element computation, assembling the matrices requires extensive numerical integration on each element. This can be conducted in parallel by distributing elements among the available nodes. In this regard, a primary domain decomposition technique using the Message Passing Interface (MPI) was implemented to assign a subset of elements to each available computing node. After performing the integration, the results of all nodes are gathered to assemble the linear system of equations. In the current implementation, an MUMPS sparse direct solver (v5.5.1, Mumps Technologies SAS, Lyon, France) [32] was used to solve the linear system. The post-processing of the results was carried out using ParaView (v5.11, Kitware Inc., New York, NY, USA) [33].

#### 2.1.3. Optimization of the Velocity Control Value

Obtaining the correct values for the parameters of a computational model can be pretty challenging and may require dedicated experimental input. In this regard, defining an efficient inverse problem can help save time and resources when estimating the unknown parameters. In this study, a dedicated in vitro experiment was set up, and the results were used in a Bayesian optimization routine [34] to calibrate the parameter A. The objective function of the inverse problem was the root-mean-square error of the difference between the predicted and experimentally obtained values of tissue growth rate over 21 days.

### 2.2. In Vitro Experiments

#### 2.2.1. Design of the Disk

In order to efficiently test a range of pore geometries and sizes, a disk was designed with a height of 2 mm and a diameter of 14 mm, fitting the well of a 24-well plate. In the disk, channels were included, with four basic cross-sections (triangle, square, hexagon, and circle) and three sizes. They were 0.5 mm, 0.7 mm, and 1 mm for the circle and hexagon and 0.7 mm, 1 mm, and 2 mm for the square and triangle, as with the latter shapes, the smallest size could not be accurately produced. Each combination of cross-sectional shape and size was repeated three times. All channels were arranged randomly on the disk, with at least 60 µm in between them. Table 1 provides a summary of the experimental setup.

#### 2.2.2. Design of the Gyroid Scaffold

Upon confirmation of the curvature-based growth principle in CaP-based scaffolds (see results section), a 3D structure was designed that allowed us to test the potential of the in silico model to predict neotissue growth in more complex geometries. Triply periodic minimal surface structures in general and gyroid structures in particular provide an environment with a well-controlled curvature and a narrow curvature distribution [24]. Gyroid structures (or triply-periodic minimal surfaces in general) have received an increasing amount of attention over the last couple of years for this reason. In addition to being interesting from a biological/mathematical perspective, they are also very manufacturing-friendly, as the geometry varies very smoothly from one layer to the next.

#### 2.2.3. Production of the Disk and Scaffold

The disks were produced through stereolithography using different CaP-based pastes from Cerhum (Liège, Belgium): pure hydroxyapatite (HAp100), pure tricalcium phosphate (TCP100), and a 60/40 mixture of the two (HAp60 TCP40), also known as BCP. Stereolithography is an additive manufacturing process that builds polymer parts in 3D by photocuring a liquid or paste. Here, the bioceramic powder was carefully mixed with organic components (polyfunctional acrylic resins and a UV photoinitiator) in order to obtain a viscous paste material with roughly 50% solid loading to be processed by SLA (Cerhum and Sirris, Liège, Belgium). During manufacturing, the suspension was spread on the working area in thin layers of 50 µm, after which UV light was projected by a digital light onto the paste surface. Subsequently, the samples were subjected to a thermal cycle (1030 °C for TCP100 and HAp60-TCP40 and 1130 °C for HAp100 for 5 h), allowing for the removal of the resin and the densification of the ceramic, as reported and discussed elsewhere [10,24,35,36,37]. After manufacturing, the parts were rinsed and ultrasonically cleaned in an 80% ethanol bath for 10 min. The same process was followed for the manufacturing of the 3D gyroid structure (HAp100).

#### 2.2.4. Cell Culture and Analysis

After production, the disks were sterilized with an autoclave at 121 °C for 15 min. Prior to the cell culture experiment, the disks were pre-wetted for 3 h with growth medium (GM) composed of Prigrow II Medium + Fetal Bovine Serum (FBS) to a final concentration of 10% + hydrocortisone to 10^−6^ mol/L and Penicillin/Streptomycin Solution to a final concentration of 1%. After prewetting, the disks were air-dried for 1 h under sterile conditions. Then, 600,000 hTERT-Immortalized Bone Mesenchymal Stromal Cells (hTERT-BMSCs, Applied Biological Materials Inc. Richmond, Canada) were drop-seeded onto each disk suspended in a 200 µL cell suspension and subsequently incubated statically for 4 h at 37 °C to facilitate cell attachment. The amount of cells was chosen to ensure a good baseline coverage of the disk with cells without having open spaces on the disk. Then, the cell-seeded scaffolds were transferred to a 24-well plate and cultured in GM for 3 weeks. The medium was refreshed three times a week. The cell viability and kinetics of the neotissue (cell+ECM) channel filling were evaluated after 10 and 21 days of in vitro culture for the different pore geometries using fluorescence microscopy imaging (Live–Dead viability/cytotoxicity staining (Invitrogen, Thermo Fisher Scientific inc., Waltham, MA, USA) and DAPI/Phalloidin). Disks were rinsed with 1 mL of PBS, incubated in the staining solution (0.5 mL of calcein AM and 2 mL of ethidium homodimer in 1 mL of PBS) for 20 min under standard cell culture conditions, and finally imaged using an Olympus IX83 inverted fluorescence microscope (Evident, Tokyo, Japan).

Similar steps were followed for the 3D structure. First, 200,000 cells were drop-seeded and allowed to attach for 3 h prior to the start of the static culture. Neotissue formation was evaluated at days 10 and 21 using contrast-enhanced nanofocus Computed Tomography (nanoCT) imaging with an 80% Hexabrix 320 solution (Guerbet, Villepinte, France) as a contrast agent (applied for 20 min) to visualize the neotissue inside the scaffold. NanoCT scans of the samples were acquired using the GE Nanotom-M (Phoenix Nanotom^®^ M, GE Measurement and Control Solutions, Billerica, MA, USA). The scaffold was scanned with a diamond–tungsten target, mode 0, a 500 msec exposure time, 1 frame average, 0 image skips, 1800 images, and a 0.2 mm aluminum filter. The constructs were scanned at a voltage of 70 kV and a current of 150 μA, resulting in a voxel size of 4 μm. 

#### 2.2.5. Image Processing

All images from the fluorescence microscopy were analyzed with ImageJ software version 1.53q for Windows (v1.53, ImageJ software, Wayne Rasband and contributors, NIH, Bethesda, MD, USA), using Bio-Format (v7.0.1, Bio-Format project, Madison, WI, USA) as a plugin for ImageJ to read and write images in the formats it supports. The image analysis provided a qualitative and quantitative measure of the filling of each channel on the disk.

CTAn (v1.18.8.0, Bruker Belgium SA, Kontich, Belgium) was used for image processing and the quantification of newly formed tissue based on automatic Otsu segmentation, 3D space closing, and a de-speckle algorithm. The percentage of neotissue was calculated in relation to the total scaffold volume. CTVox (v3.3.0, Bruker Belgium SA, Kontich, Belgium) was used to create 3D visualization.

#### 2.2.6. Statistical Analysis

All data from the quantitative processing of the fluorescence microscopy images were statistically analyzed using GraphPad Prism software version 8.2.1 for Windows (GraphPad Software, San Diego, CA, USA). To compare multiple groups’ means with three repeats, a statistical analysis of the results was performed by a two-way analysis of variance (ANOVA), followed by post hoc tests (Tukey’s multiple comparison test). Significant levels are reported as follows: * *p* < 0.05, ** *p* < 0.01, *** *p* < 0.001, and **** *p* < 0.0001.

## 3. Results

### 3.1. Analysis of In Vitro Cell Behavior

**Figure 2:**
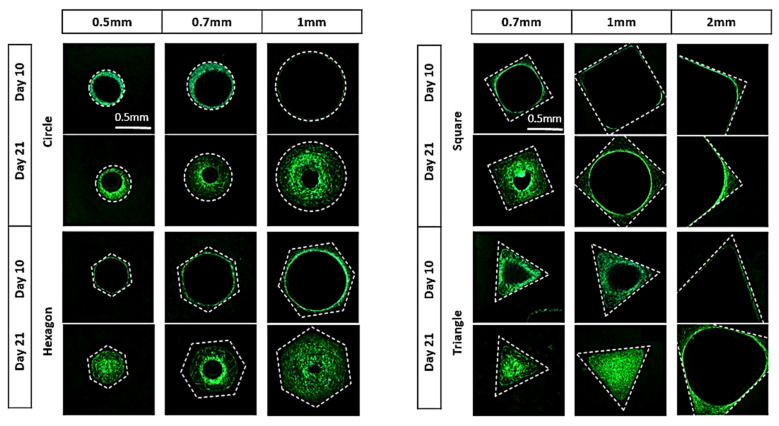
Neotissue growth results in the different channels for HAp disks (representative images) for the different channel shapes and diameters over time. Looking vertically, it is evident that for every shape and size, curvature-driven neotissue formation is taking place over time. Scale bar (0.5 mm) is the same for all panels.

**Figure 3:**
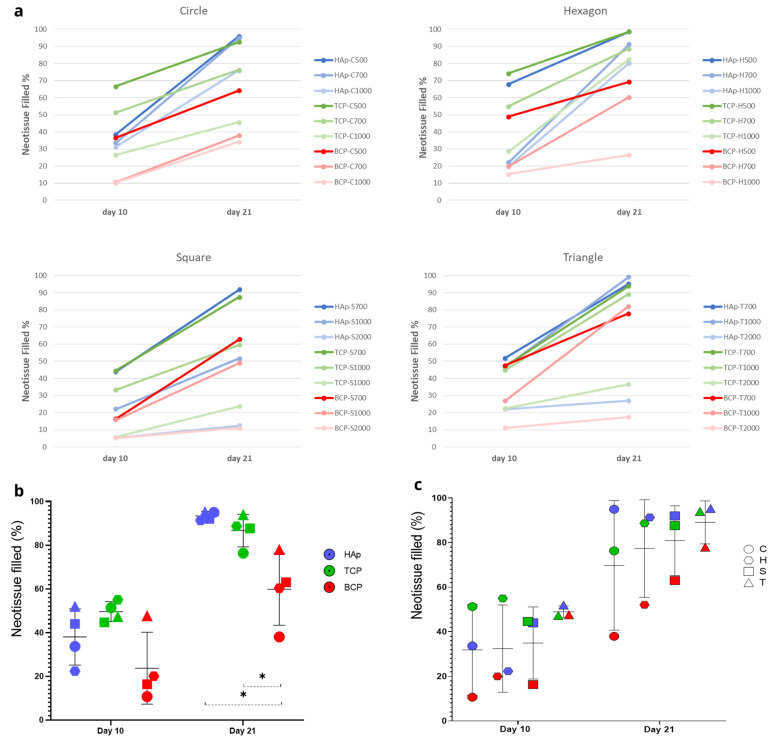
Quantification of experimental results. (**a**) Percentage of channel cross-section filled with neotissue after 10 and 21 days for the different channel shapes, shown as the mean. The labels in the legend refer to the material used (HAp, TCP, BCP), the shape (C: circle, H: hexagon, S: square, T: triangle), and the channel diameter in micrometers. (**b**) Percentage of channels with a diameter of 0.7 mm filled with neotissue after 10 and 21 days comparing different CaP biomaterials, shown as the mean of various shapes ± SD, and (**c**) percentage of channels with a diameter of 0.7 mm filled with neotissue after 10 and 21 days comparing different shapes, shown as the mean of various biomaterials ± SD. Statistical significance is calculated by two-way ANOVA test; * *p* < 0.05.

**Figure 4:**
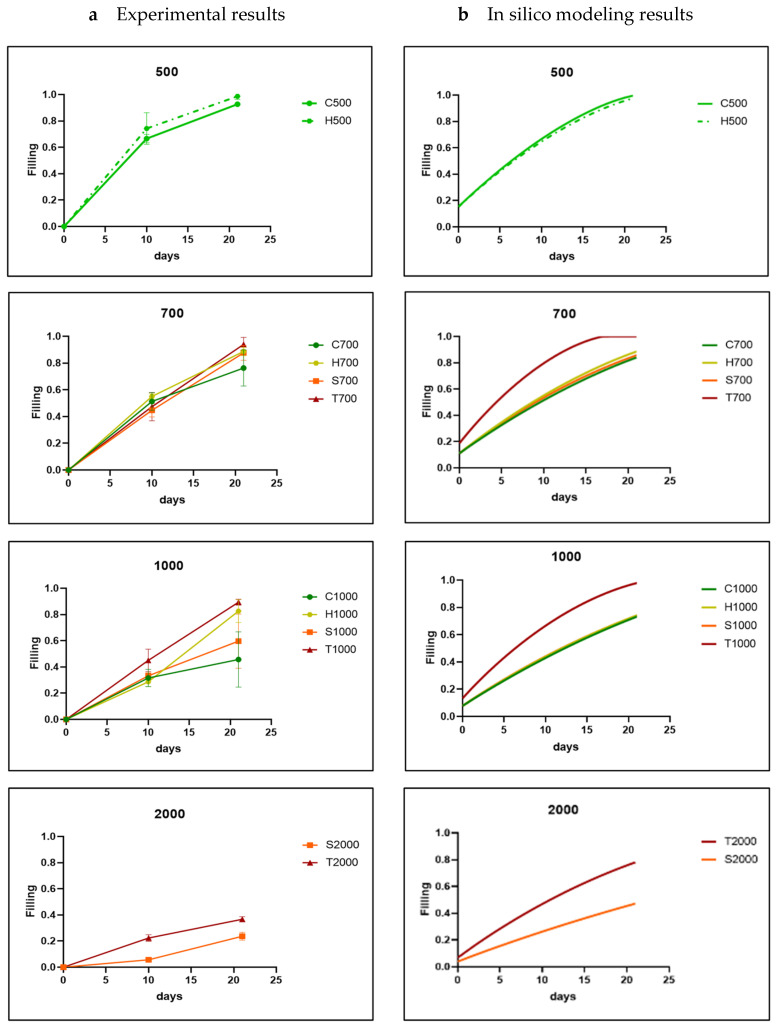
Comparison between experimental results (**a**) and in silico results (**b**) for each channel size (parameter A was fixed at 0.3 during Bayesian optimization) for HAp disks. The shapes are labelled by a letter (T: triangle; S: square; H: hexagon; C: circle) and a number indicating the channel diameter in micrometers. The experimental data are shown as mean ± SD.

**Figure A1:**
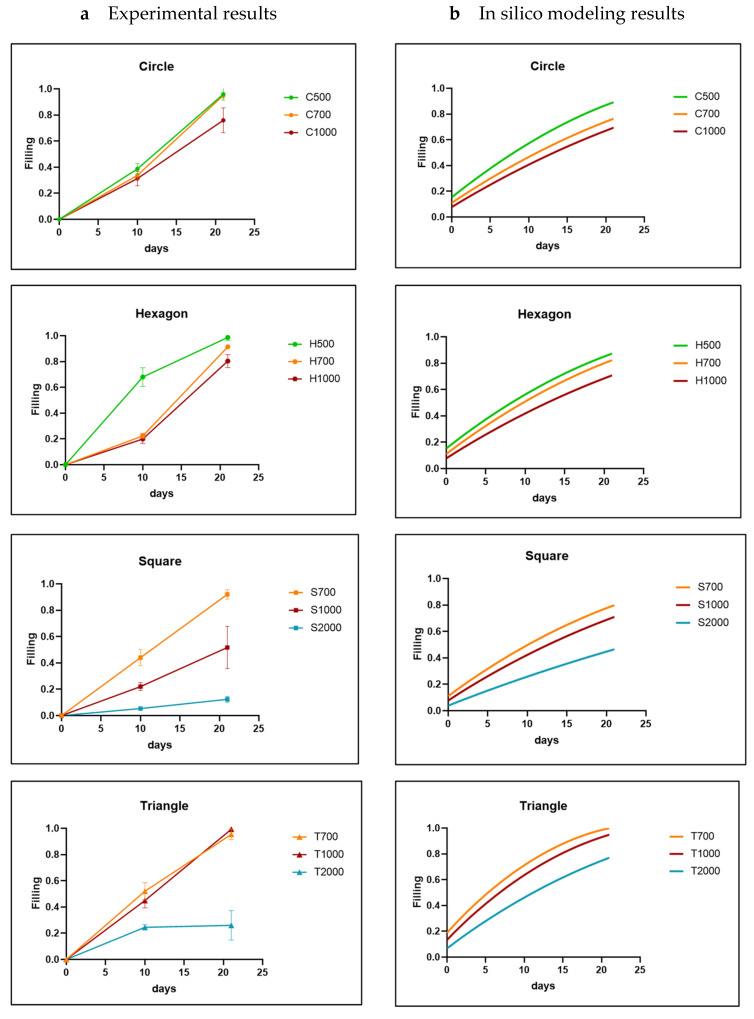
Comparison between experimental results (**a**) and in silico results (**b**) for all channel shapes for TCP disks. The parameter A was fixed at 0.01 during Bayesian optimization. The shapes are labelled by a letter (T: triangle; S: square; H: hexagon; C: circle) and a number indicating the channel diameter in micrometers. The experimental data are shown as the mean ± SD.

**Figure A2:**
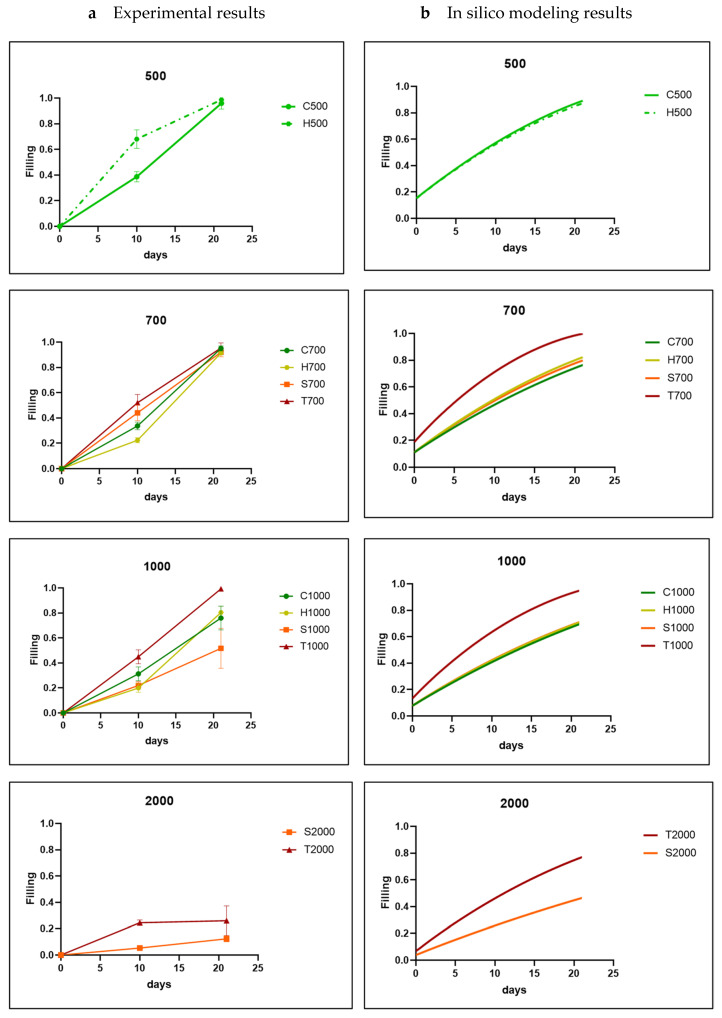
Comparison between experimental results (**a**) and in silico results (**b**) for all channel sizes for TCP disks. The parameter A was fixed at 0.01 during Bayesian optimization. The shapes are labelled by a letter (T: triangle; S: square; H: hexagon; C: circle) and a number indicating the channel diameter in micrometers. The experimental data are shown as the mean ± SD.

**Figure A3:**
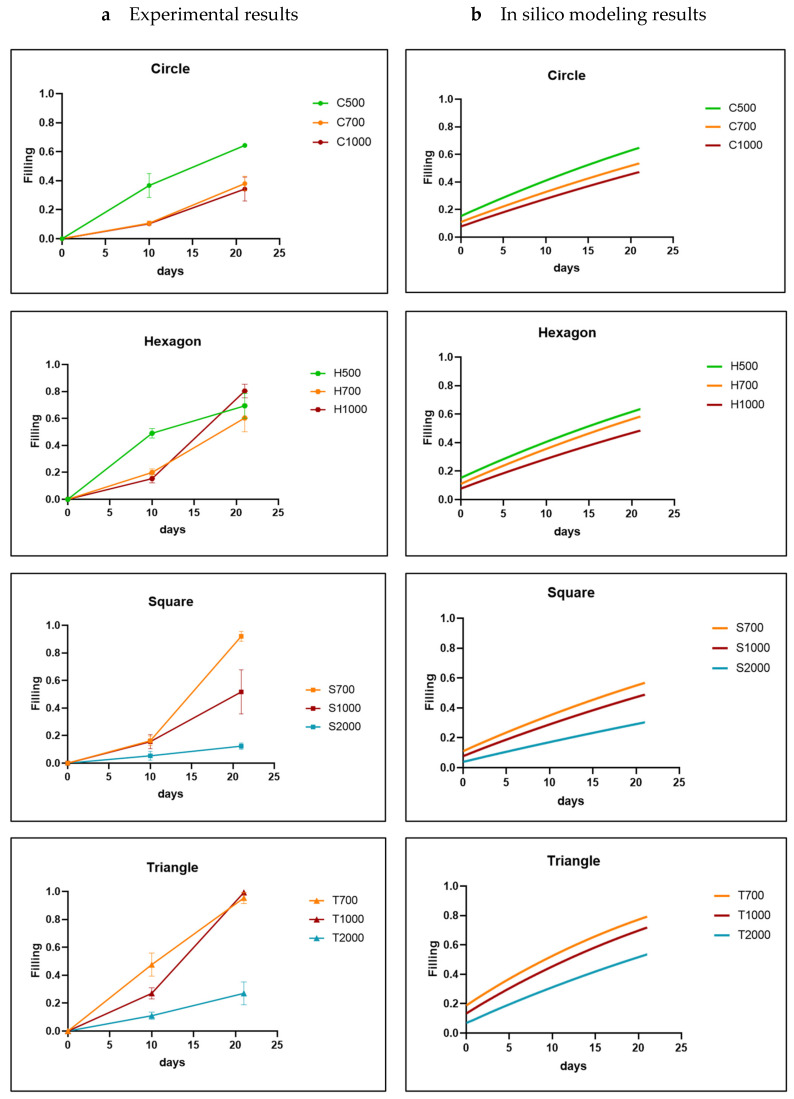
Comparison between experimental results (**a**) and in silico results (**b**) for all channel shapes for BCP disks. The parameter A was fixed at 0.001 during Bayesian optimization. The shapes are labelled by a letter (T: triangle; S: square, H: hexagon; C: circle) and a number indicating the channel diameter in micrometers. The experimental data are shown as the mean ± SD.

**Figure A4:**
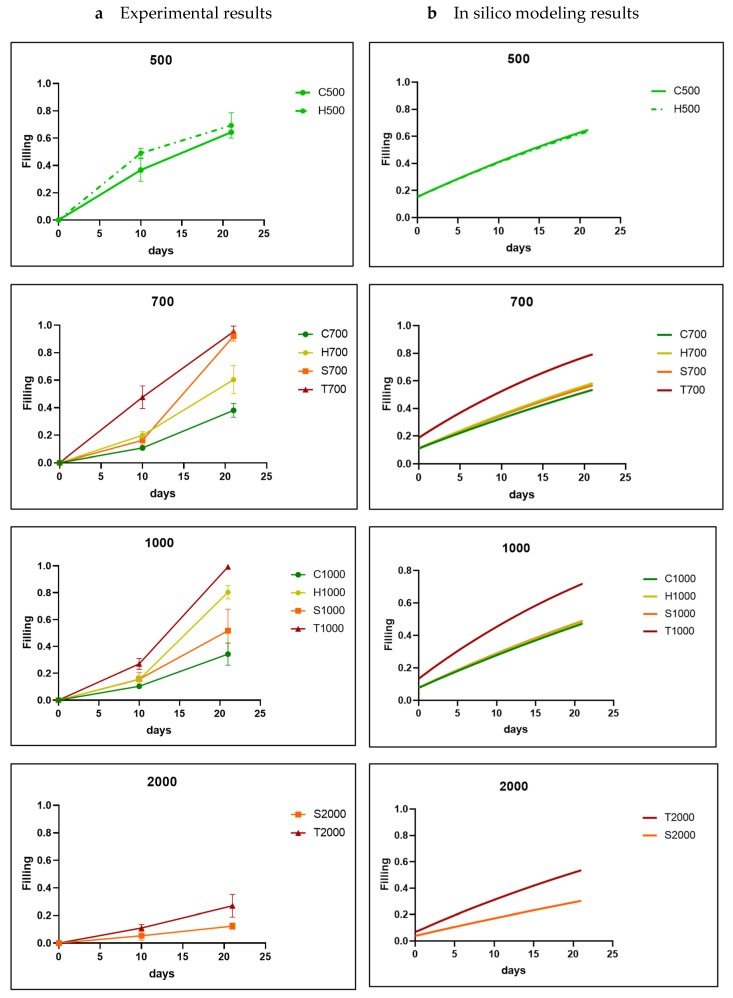
Comparison between experimental results (**a**) and in silico results (**b**) for all channel sizes for BCP disks. The parameter A was fixed at 0.001 during Bayesian optimization. The shapes are labelled by a letter (T: triangle; S: square; H: hexagon; C: circle) and a number indicating the channel diameter in micrometers. The experimental data are shown as the mean ± SD.

The percentage of neotissue formed within the channels was calculated using image processing on fluorescent images (Figure 2). The Live/Dead staining showed the good biocompatibility of the produced CaP disks with the hTERT-BMSCs. The fluorescence images revealed a viable cell population for all pore channels on both time points with a greater abundance for day 21 compared to day 10. Cells were seen to attach to the top surface of the disk as well as the pore walls. The pattern of neotissue growth in the channels, particularly for the triangle, square, and hexagon shapes, demonstrates that neotissue growth indeed starts in the areas of the highest curvature, ultimately forming a circular growth boundary. Subsequently, neotissue continues to grow towards the center of the channel, gradually filling it up. This was observed to happen regardless of the shape and size of the initial channel or the material used, confirming the curvature-based hypothesis for the tested materials. The results of the quantification of neotissue formed for different channel cross-sectional shapes and sizes and different types of CaP biomaterials on days 10 and 21 are shown in Figure 3 and in Table A1 and Figure A1, Figure A2, Figure A3 and Figure A4 in Appendix A, respectively. When comparing the different materials (using the channels with 0.7 mm and 1 mm diameters as examples), the experiments demonstrated that the results for HAp (0.7 mm: 38% on day 10 to 93.42% on day 21; 1 mm: 30% on day 10 to 76.83% on day 21) and TCP (0.7 mm: 49.58% on day 10 to 86.67% on day 21; 1 mm: 33.42% on day 10 to 69.33% on day 21) were not significantly different; however, the BCP results (0.7 mm: 23.67% on day 10 to 59.83% on day 21; 1 mm: 17.08% on day 10 to 48.08% on day 21) were significantly lower on day 21 (Figure 3b). Comparing the pore shapes, the triangles mostly showed faster growth than squares, hexagons, and circles (Figure 3c and Figure 4a), although the influence of the material and pore size confounded the results. For the largest sizes (2 mm in the triangle and square channels), the growth rate was strongly reduced compared to all others, with limited neotissue formation present in the corners, though the circularization of the neotissue interface was still visible. 

### 3.2. In Silico Modeling

**Figure 5:**
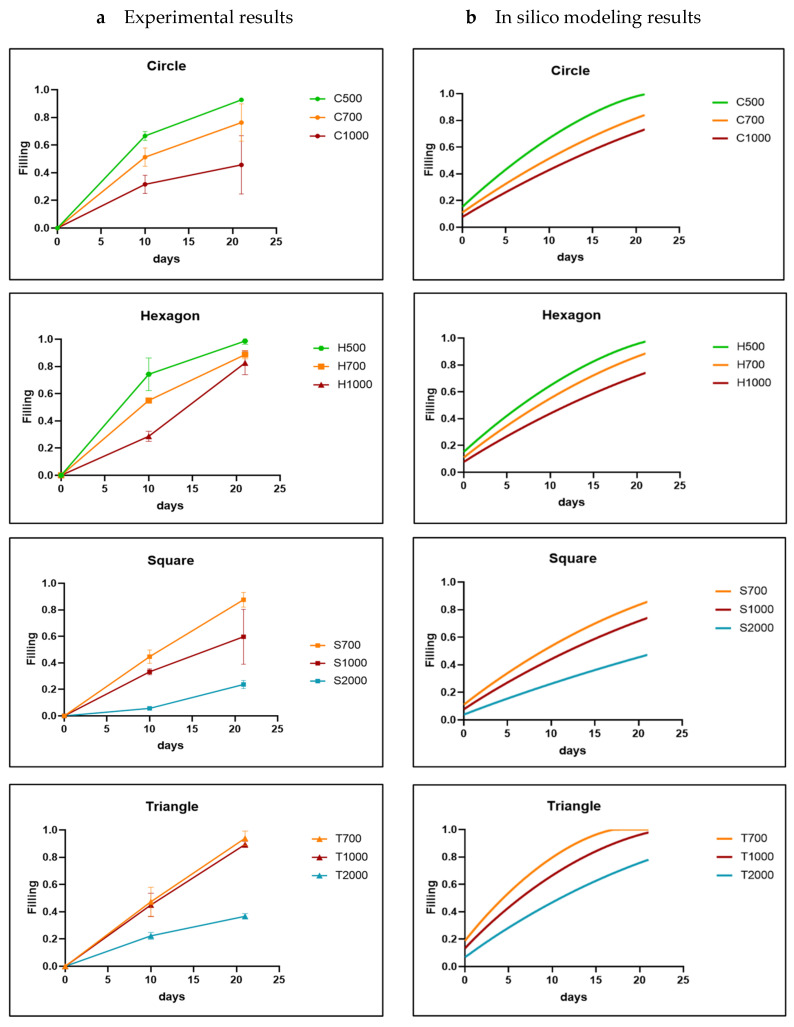
Comparison between experimental results (**a**) and in silico results (**b**) for each channel shape (parameter A was fixed at 0.3 during Bayesian optimization) for HAp disks. The shapes are labeled by a letter (T: triangle; S: square; H: hexagon; C: circle) and a number indicating the channel diameter in micrometer. The experimental data are shown as mean ± SD.

As the experimental results confirmed the in silico model’s basic premise of curvature-based neotissue growth, qualitatively, the simulation results largely corresponded to the experimental ones. Bayesian optimization was used as indicated in the Methods section in order to calibrate the model parameter A for all materials, shapes and sizes and was ultimately fixed at 0.3 for the HAp disks, 0.01 for the TCP, and 0.001 for the BCP disks. The optimization led to a good quantitative correspondence between the experimental and simulation results, shown in relation to channel size (Figure 4) and channel shape (Figure 5). The simulation results showed, as expected from the curvature-based principles, that increasing the channel diameter decreased the neotissue growth rate (Figure 4). For the channel sizes 0.5 mm and 0.7 mm, all the shapes reached high filling percentages on day 21. However, especially for 0.5 mm, the experimental time points did not allow us to assess the exact time point at which 100% filling was reached. Hence, this could explain the qualitative difference in filling rates between the experiments and simulations, with the filling tendency appearing as a polyline in the experimental result and a smoother line in the simulations. The hexagon shows the fastest neotissue growth across all sizes, whereas for the smallest sizes (0.7 mm and 1 mm), the triangular channel fills up fastest both in the experiments and the simulations due to the curvature being highest in those channels and the neotissue growing inward from the corners being more likely to establish contact quickly. For the size 1 mm, the triangle was still the fastest-growing one, almost reaching 100% filling on day 21, followed by the circle and hexagon, which reached about 60% filling on day 21. The square was relatively slow, and the final filling rate was about 40%. For the size 2 mm, the filling rate of the four basic shapes did not exceed 20%.

### 3.3. Model-Informed 3D Scaffold Design and Validation

**Figure 6:**
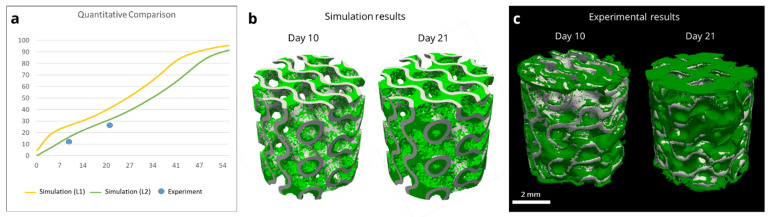
Comparison between in vitro experiment and simulations for 3D HAp gyroid structure. (**a**) Quantification of the neotissue formation (% of filling as a function of time (days)) in the experiment (points) and simulations (full line). L1 = initial thickness of neotissue layer, 10 µm; L2 = initial thickness of neotissue layer, 1 µm. (**b**) Quantitative view of simulation results on day 10 and day 21. (**c**) Contrast-enhanced nanoCT images of in vitro experiments on day 10 and day 21, with neotissue in green pseudo-color.

Based on the results obtained with the basic geometries, neotissue growth in a 3D HAp structure was predicted and experimentally assessed to provide a validation step. A triply-periodic minimal surface structure (gyroid) was designed with a 0.2 mm wall thickness and 0.9 mm pore size (Figure 1d) to respect manufacturing constraints. Due to differences in the initial seeding densities between the experimental disc and 3D structure experiments, different values of the thickness of the initial cell layer were tested (10 µm (L1 in Figure 6a) and 1 µm (L2 in Figure 6a), respectively), as seeding at a non-confluent density was followed by a period of mostly 2D growth before starting growth in the third dimension, leading to an overall reduction in the speed of neotissue formation (Figure 6a,b). In vitro experiments under static conditions in growth medium were executed for the designed gyroid structure, produced in HAp, and analyzed using contrast-enhanced nanoCT imaging (with Hexabrix as a contrast agent) (Figure 6c). The quantitative comparison demonstrated a similar trend in neotissue growth between day 10 and day 21, illustrating the potential of the model to be used as a tool to design 3D bone tissue engineering scaffolds. 

## 4. Discussion

Optimizing the scaffold shape with respect to cell (in)growth remains an open challenge in tissue engineering. With additive manufacturing, not only material composition and overall porosity but also the microarchitecture can be designed and accurately produced. The present model builds on previous work for simulating neotissue growth in titanium additively manufactured scaffolds [22] in order to investigate neotissue growth in calcium-phosphate-based scaffolds. First, a dedicated 2D+ in vitro experiment was designed, allowing us to qualitatively and quantitatively compare the influence of channel shape and size for different CaP materials. The final calibrated model was then used to predict neotissue growth on a 3D gyroid-based scaffold, showing adequate agreement between the simulation results and the in vitro experiments. The most important contribution of this study is the application of the neotissue growth model to CaP additively manufactured scaffolds, moving from basic shapes and 2D+ substrates to experimentally validated complex 3D structures. 

A Bayesian approach was followed for calibrating the computational-intensive model [38] since it minimizes the number of optimization iterations, during each of which the computational model should run at least once. Since evaluating the objective function is expensive, a Bayesian optimization routine considers the previous iterations to choose the following values by constructing a probability tree of the objective function, acting as a surrogate model, which makes the selected approach more efficient than gradient-based or fully stochastic methods [34]. The probability model is a conditional probability, p (score parameters), which gets updated by the optimization algorithm during each iteration by incorporating newly obtained results. This operation was carried out by Sequential Model-Based Optimization (SMBO) methods, which need fewer optimization iterations than methods relying on a random selection of values (stochastic methods) or approaches needing an evaluation of the objective function at least twice (gradient-based methods) [39].

Regardless of the basic shape and channel size tested, the neotissue growth showed the hallmarks of curvature-based growth, including the circularization of the neotissue-void interface taking place over time and the neotissue growth speed decreasing for the larger channel sizes. For all the materials tested, the triangular shape demonstrated the fastest growth with the lowest variability compared to the other channel geometries of the same size. This might appear to contrast with our previous study, where, when testing basic shapes in titanium scaffolds, the triangle performed worst in terms of speed [22]. However, in that study, the parameter d was chosen as the diameter of the inscribed circle rather than the side of the triangle, as was done here (Figure 1b), leading to a substantially larger surface compared to the other shapes (~30%) with longer straight edges between the corners and hence a slower filling. The results of this study are in agreement with other reports using a dimensionalization similar to the one used here [20]. For the smallest diameter channels (500 µm in the hexagon and circle), complete filling was reached during the experiment in between the first and second observation time points, explaining the experimentally observed change in growth speed between both points. Not knowing the exact point of filling, the simulations were unable to account for it accurately, leading to a smoother behavior in the simulation results compared to the experimental observations. 

Extending the use of the model from basic shapes towards 3D structures for the same materials and experimental settings is a strong point of this study. For the 2D+ set-up, cells were seeded at a density close to confluency to speed up the onset of growth inside the channels. For the 3D structure, a lower initial density was chosen, moving towards densities more typically used in tissue engineering applications [40]. This meant that the initial phase of the neotissue growth was mostly driven by the growth of cells onto the substrate [41]. As this type of growth is not captured by the current model, it was simulated by lowering the initial thickness of the cell layer to 1µm. This resulted in slower predicted neotissue growth in the initial phase, followed by a neotissue growth rate and final filling density similar to those obtained for a higher initial cell layer, in line with the experimental observations (Figure 6a). This second phase of neotissue growth is characterized by cells growing on top of the extracellular matrix they have produced themselves, as described in [41]. The gyroid structure used in this study (defined by its pore size and wall thickness) was the result of an in silico study in the context of oral bone regeneration, balancing the need for rapid neotissue (in)growth, the need for a high neotissue-to-biomaterial ratio, and the constraints imposed by the additive manufacturing process. The gyroid structure was tested for its capacity to induce in vivo bone formation in a cranial augmentation model (implantation without seeded cells), showing the superiority of the design over the clinically used gold standard and a lattice structure control [10]. 

Compared to our own previous work [22,42], moving from titanium to CaP-based materials led to a decrease in the neotissue growth rate. This might be related to the active nature of the CaP material, which could be shifting the balance from the proliferation of the progenitor cells towards their early differentiation [43] or to the difference in surface composition and topography [44]. Both factors might also provide additional insight into the obtained experimental differences for the different materials that were tested. A wide range of in vitro and in vivo studies have been reported in the literature with the different calcium phosphate materials used in this study (reviewed extensively in [45,46,47,48,49,50,51]). These reports describe how differences in composition, manufacturing techniques, sintering temperatures, surface treatments, etc., result in differences in terms of (amongst others) mechanical properties, dissolution rates, biological activity, and bone formation potential. Added to this are the effects that the in vitro and in vivo conditions themselves have on the experimental results (e.g., the same materials respond differently in different animal models [50]). Confirmation of the possible causes explaining the observed differences between the materials in this study could be obtained from additional biological experiments involving gene expression analysis on the cultured cells in the neotissue or material tests such as X-ray diffraction to analyze material decomposition; however, this falls outside of the scope of this study. 

This study provides additional experimental and numerical support for the current research focus on triply-periodic minimal surfaces in bone tissue engineering. This focus was inspired by the development of the relatively new field of curvature-based biology (see [19] and references within). In this field, the mechanistic underpinnings of the effect of local curvature on neotissue growth (linked to intercellular tensile forces) have been investigated in a range of materials and applications [21,25,26,41,52]. On the other hand, in the tissue engineering field, many studies address either theoretical aspects of the description of 3D structures [53] or focus on particular mechanical or mass transport [54,55,56]. This study sits at the interface between the aforementioned approaches, using a combined in vitro–in silico approach with a focus on the biological outcome. As such, it provides a clear basis for the further testing of these structures in in vivo settings [10]. 

## 5. Conclusions

In this study, a curvature-based tissue growth model was adapted for use in calcium phosphate 3D additively manufactured structures. After model calibration by a coupled in silico–in vitro approach, the final model’s potential for simulating neotissue growth was demonstrated on a 3D gyroid scaffold. The in silico framework presented in this study has demonstrated its ability to be used as a tool for designing improved bone tissue engineering scaffolds and can easily be extended with additional design features for other applications in the future.

## Figures and Tables

**Table 1:** Disk design, manufacturing parameters, and experimental variables considered for the in vitro experiment in this study.

Property	Description
Pore shapes	Triangle, square, hexagon, and circle
Pore size	0.5 mm, 0.7 mm, 1 mm, and 2 mm
Distribution	Randomly
Materials	Hydroxyapatite (HAp, 100%), Tricalcium phosphate (TCP, 100%), and mixed HAp 60%–TCP 40% pastes (supplier Cerhum)
Sintering temperature	1030 °C for TCP100 and HAp60-TCP40 and 1130 °C for HAp100
Time points analysis	10 days and 21 days
Cell type	Human telomerase reverse transcriptase-immortalized bone marrow mesenchymal stem cells (hTERT-BMMSCs)

## Data Availability

Quantified data are contained within the article and supplementary materials. All raw images and software can be made available upon request.

## Appendix A

**Table A1:** Raw numbers and averages of the neotissue filling percentages for the different time points (10 and 21), materials (HAp, TCP, BCP), channel shapes (C: circle; H: hexagon; S: square; T: triangle), and channel sizes (500: 0.5 mm; 700: 0.7 mm; 1000: 1 mm; 2000: 2 mm).

[%]	S1	S2	S3	Average	S1	S2	S3	Average	S1	S2	S3	Average	S1	S2	S3	Average
	HAp-C500				HAp-C700				HAp-C1000							
day 10	43	35	38	38.67	37	31	33	33.67	34	35	25	31.33				
day 21	97	91	100	96.00	97	95	93	95.00	86	75	67	76.00				
	HAp-H500				HAp-H700				HAp-H1000							
day 10	62	66	76	68.00	24	20	23	22.33	18	24	18	20.00				
day 21	100	96	100	98.67	93	90	91	91.33	75	81	85	80.33				
					HAp-S700				HAp-S1000				HAp-S2000			
day 10					51	39	42	44.00	19	25	22	22.00	5	5	6	5.33
day 21					96	91	89	92.00	53	67	35	51.67	15	11	11	12.33
					HAp-T700				HAp-T1000				HAp-T2000			
day 10					59	46	51	52.00	49	41	50	46.67	26	23	17	22.00
day 21					93	100	93	95.33	98	100	100	99.33	34	18	29	27.00
	TCP-C500				TCP-C700				TCP-C1000							
day 10	63	69	68	66.67	53	44	57	51.33	30	24	26	26.67				
day 21	93	93	92	92.67	69	68	92	76.33	32	70	35	45.67				
	TCP-H500				TCP-H700				TCP-H1000							
day 10	63	87	73	74.33	57	53	55	55.00	26	33	27	28.67				
day 21	96	100	100	98.67	86	92	88	88.67	90	73	85	82.67				
					TCP-S700				TCP-S1000				TCP-S2000			
day 10					40	50	44	44.67	36	32	32	33.33	7	5	5	5.67
day 21					85	94	84	87.67	41	82	56	59.67	21	27	23	23.67
					TCP-T700				TCP-T1000				TCP-T2000			
day 10					36	49	57	47.33	35	50	50	45.00	20	22	25	22.33
day 21					92	100	90	94.00	88	88	92	89.33	35	36	39	36.67
	BCP-C500				BCP-C700				BCP-C1000							
day 10	46	30	34	36.67	12	9	11	10.67	10	11	10	10.33				
day 21	63	65	65	64.33	33	43	38	38.00	41	37	25	34.33				
	BCP-H500				BCP-H700				BCP-H1000							
day 10	45	52	50	49.00	21	22	17	20.00	13	14	19	15.33				
day 21	65	80	63	69.33	69	49	63	60.33	26	27	27	26.67				
					BCP-S700				BCP-S1000				BCP-S2000			
day 10					20	16	13	16.33	17	20	10	15.67	3	9	4	5.33
day 21					79	44	66	63.00	42	60	45	49.00	13	11	9	11.00
					BCP-T700				BCP-T1000				BCP-T2000			
day 10					57	41	45	47.67	23	27	31	27.00	9	10	14	11.00
day 21					85	65	84	78.00	84	78	85	82.33	21	16	15	17.33
